# Strategic Adaptive Management for Transparency, Accountability and Learning: Insights from More Than a Quarter Century of Practice

**DOI:** 10.1007/s00267-025-02272-5

**Published:** 2025-09-04

**Authors:** Dirk J. Roux, Richard T. Kingsford, Jessica Cockburn

**Affiliations:** 1 Scientific Services, South African National Parks, Knysna, Garden Route South Africa; 2 Sustainability Research Unit & CNRS-NMU International Research Laboratory REHABS, Nelson Mandela University, George, South Africa; 3 Centre for Ecosystem Science, School of Biological, Earth and Environmental Sciences, University of New South Wales, Sydney, Australia; 4 Department of Environmental Science, Rhodes University, Makhanda (Grahamstown), South Africa

**Keywords:** Adaptive management, Bibliographic analysis, Case study, Social-ecological systems, Strengths and weaknesses

## Abstract

Adaptive management has long been advocated as a framework of choice for addressing the complexities and uncertainties of natural resource management. Despite its theoretical appeal, successful implementation remains elusive, with many documented barriers and limited operational examples. This paper examines Strategic Adaptive Management (SAM), a long-running adaptive management program originating from the Kruger National Park in South Africa. SAM’s formulation in the 1990s drew on principles from value-based business planning and adaptive management, emphasizing the co-defining of a desired state or aspirational outcome that incorporates societal values, management pragmatism and scientific rigor. Following a case study approach, we analyze SAM’s conceptual evolution and geographic spread through a bibliographic synthesis including operational and trialed case examples spanning rivers, protected areas and rural landscapes. We identify three parallel streams of development, in South Africa’s national parks, Australia and the Eastern Cape province of South Africa. Experiences, including strengths and weaknesses, from these streams were used to characterize present-day SAM and its iterative cycles of envisioning a desired state, considering management options, implementing actions, reviewing outcomes and adapting, alongside omnipresent co-learning and reflection. Additionally, the paper contextualizes SAM within the broad application of adaptive management, including active- and passive adaptive management, adaptive co-management and Conservation Standards, highlighting similarities and opportunities for cross-learning. Our synthesis underscores SAM’s transparency, scalability and value in fostering stakeholder collaboration and co-learning across diverse environmental contexts. SAM offers a robust framework for achieving strategic, vision-oriented management amidst uncertainty, even with implementation challenges.

## Introduction

Adaptive management is widely promoted as the most suitable and defendable approach to natural resource management, promoting learning in social-ecological systems characterized by high uncertainty. Formulated in the 1970s (Holling 1978), adaptive management incorporates dynamic states of natural resource systems, influenced by fluctuating environmental conditions (Williams 2011). System responses to changing conditions and actions, including anthropogenic influences, are highly uncertain, requiring ongoing learning (Allen et al. 2011). Adaptive management uses systematic and purposeful learning about a system and its response to drivers and threats to improve management. This typically requires: identifying a management goal or vision and defining management objectives with stakeholders; linking these to potential management actions, which can be experimentally compared or scenario tested; implementing selected management actions; monitoring system responses to management interventions; changing practice where required; documenting learning and; repeating the cycle (Rist et al. 2013a; Westgate et al. 2013).

Theory and practice of adaptive management is expanding, supported by increasing literature, including seminal works (e.g. Walters 1986; Lee 1993; Gunderson et al. 1995; Berkes et al. 2003). Despite conceptual simplicity, intuitive appeal and widespread enthusiasm, adaptive management has proven difficult to implement (Walters 1997; Allen and Gunderson 2011; Rist et al. 2013a), with operational examples being extremely rare (Lee 1999; Allen et al. 2011; Westgate et al. 2013). Commonly-mentioned implementation challenges include the inherent complexity of social-ecological settings that engender intractable problems and stakeholder conflict (Lee 1999); cost of adaptive experimentation, monitoring and public consultation (Walters 1997; Westgate et al. 2013); institutional and legal frameworks without necessary flexibility (Walker et al. 2004; Novellie et al. 2016); management, policy, and funding paradigms that favor reactive rather than proactive approaches (Walters 1997; Schreiber et al. 2004); and insufficient success stories (Lee 1999; Walters 2007). The many barriers to implementation remain germane to all management approaches (Rist et al. 2013b), with ongoing discussion (e.g. see Månsson et al. 2023), almost 50 years since adaptive management was first proposed.

Here we contribute an example of an operational adaptive management program, with a long-running track record, in spite of implementation challenges: Strategic Adaptive Management (SAM). SAM has its roots in the Kruger National Park (KNP) and a track record of more than 25 years. It focuses on driving responsible management of natural resources, with the key elements of any rigorous approach. It has spread beyond KNP and South Africa (notably to Australia) and is increasingly applied to different management issues and contexts. In spite of this, SAM has remained relatively unnoticed outside of its practitioner communities, without reference in prominent reviews (e.g. Westgate et al. 2013; Månsson et al. 2023) and books (e.g. Allen and Garmestani 2015) on adaptive management.

We used a case study approach to explore the development and implementation of SAM through the lens of adaptive management, focusing on its literature profile, conceptualization and application. We also focus on recent developments in three semi-autonomous parallel ‘streams’ of SAM development to highlight strengths and challenges, provide an up-to-date characterization of SAM practice, and reflect on how SAM aligns with other adaptive management approaches. The SAM approach produces visions and hierarchies of objectives beyond the usual ecological or natural resource management emphasis typical in adaptive management. It also creates learning environments by encouraging caring, trust building, regular reflection and knowledge sharing, essential to effective implementation of SAM (Palmer et al. 2022; Weaver et al. 2023; Rosenberg et al. 2023).

As practitioner-theorists, we have hands-on experience of SAM, engaged across all three streams. Our involvement in SAM practice and networks made us relatively familiar with its literature and undocumented history and developments, benefitting our aim of profiling SAM and chronicling its ‘intellectual legacy’.

## Approach Followed

We followed a case study approach, which is an in-depth exploration of a particular project, policy, institution or program (Simons 2009; Schwandt and Gates 2018). This chronicles the case study to reveal and share new understanding.

There are many and diverse ways for conducting case study research, with an emphasis on diverse sources of evidence. We combined a bibliographic analysis (Supplement 1), a historical review of the literature, and personal experience. For the bibliographic analysis, we aimed to identify the set of publications contributing either seminally or more nuanced to SAM development. First, we searched Web of Science (WoS) and Scopus (on 8 February 2024) for “strategic adaptive management” in the titles, abstracts and keywords of publications up to the end of 2023. WoS delivered 43 publications and Scopus 44, with 34 publications shared across the two sources. We checked their validity, excluding non-related accidental inclusions (8), those with a cursory reference to SAM (5), a duplicate and one popular article, leaving 38 distinct publications (34 WoS and 32 Scopus). We then added 36 journal articles, commentaries, book chapters and reports, not retrieved through searches. These made up two groups, collected throughout: (a) those we knew were essential in explaining SAM, from our personal knowledge and engaging with others in the SAM network (31); and (b) those revealed through snowball reading (5). Our two-step process resulted in a final list of 74 publications for review: journal articles (56), reports (10); book chapters (5); journal commentaries (2); and an editorial (1) (Supplement 1).

**Fig. 1:**
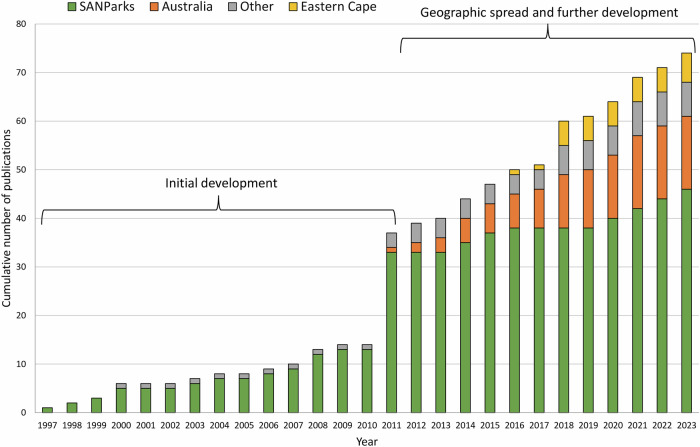
Cumulative publications on Strategic Adaptive Management (SAM), 1997–2023 (*n* = 74), delineated into two phases (initial and extended development) and separated into SANParks (*n* = 46), Australian (*n* = 15) and Eastern Cape (*n* = 6) ‘streams’, with remaining papers categorized as “other” (*n* = 7). The marked increase in 2011 resulted from a published 18-article feature on the development of SAM in SANParks (see Supplement 1)

We attributed each publication with nationality of the lead author (as per primary affiliation), parallel stream, and main application or theme (e.g. river conservation, protected area management or learning). We also organized publications chronologically through two development phases of SAM: initial development (1997–2011); and, geographic spread and further development (2012–2023) (Fig. 1).

Three streams represented three geographically distinct SAM foci, spanning trialing to operational, with different institutional and application contexts. These streams contributed 67 of the 74 publications (90.5%) (Fig. 1). The different streams provided a source of richness for characterizing present-day SAM. We also used our first-hand experiences to highlight strengths and weaknesses experienced, identified from critical discussion among the authors.

## Conceptualization, Development and Spread of SAM

### Conceptualization of SAM

SAM was an adaptation of good business planning and management (e.g. Keeney 1992), combined with adaptive management principles and applied serendipitously to complex ecosystems in Kruger National Park. In 1988, the multi-stakeholder Kruger National Park Rivers Research Program was launched to support effective management of the perennial rivers flowing through KNP (Breen et al. 2000). These rivers originate outside the park, where upstream anthropogenic threats, including agriculture and mining, were reducing flows and degrading water quality within the park. A decision-support system was needed for consultative management among managers, researchers and other stakeholders outside the park. This led to a seminal publication (Rogers and Bestbier 1997), supported by a journal article (Rogers and Biggs 1999) and two commentaries (Rogers 1998; Rogers et al. 2000b), coining the term ‘strategic adaptive management’ and articulating its early design criteria (Table 1). ‘Strategic’ emphasized a future forward-looking focus, often articulated by a vision.

**Table 1:** Four key design criteria that informed the conceptualization of Strategic Adaptive Management (Rogers and Bestbier 1997; Rogers 1998; Rogers and Biggs 1999)

Criterion	Rationale
*1. A framework supporting lasting science-management partnership*	This acknowledged the existence of divergent and often incompatible operating philosophies and procedures of managers and scientists, requiring a workable and durable partnership. “The purpose of the partnership should be to (a) develop [sufficient] consensus on institutional purpose, culture, and structure, and (b) to neutralize the adverse consequences of divergent operating philosophies and reward systems” (Rogers 1998, page 2). An important goal of the partnership was that managers help direct research and that directed research informs management.
*2. Commitment to a ‘desired state’ (variously referred to as desired future state/condition or vision), developed by consensus*	Managers need a plan to commit resources. “Coming to consensus on the job to be done and the goals to be achieved” (Rogers 1998, page 2) focuses managers and scientists, preventing the traps of reactive crisis management and/or endless ad hoc actions. The desired state provides the basis for strategic, rather than reactive management, although reactive management is still needed sometimes.
*3. Incorporate societal values, management realities and scientific rigor in the desired state*	Desired states necessarily need to have social and ecological dimensions. An ‘objectives hierarchy’ combines these by *beginning* with articulating a set of common values, crafting a value-based statement of intent (or vision), setting social and ecological objectives, and ending with measurable targets or indicators, relevant to scientifically defensible management.
*4. Pragmatism is essential for setting objectives and identification of indicators to monitor outcomes*	Pragmatic considerations drive decision-making and choice for managers, not best-possible or technical superior options (Rogers 1998). All objectives require an indicator to track and audit outcomes. Scenarios are useful and desirable. If the expertise for modeling scenarios or endpoints is not readily available, “creative and strategic thinking” is needed to determine the pathway to identified outcomes.

An ‘objectives hierarchy’ (Criterion 3, Table 1) was, and still is, a defining feature of SAM (Rogers and Bestbier 1997; Rogers and Biggs 1999), serving as a ‘boundary object’ that enables scientists and managers to connect and forge a partnership, and providing a transparent record of direction. It represents a theory of change, linking the agreed vision to jointly mapped pathways to achieve desired outcomes. Importantly, this future ‘desired state’ (Criterion 2, Table 1) did not imply a fixed equilibrium state but explicitly allowed for spatial and temporal variability, which is representative of real systems and accommodating of diverse stakeholders (Kevin Rogers, personal communication, November 2024).

**Fig. 2:**
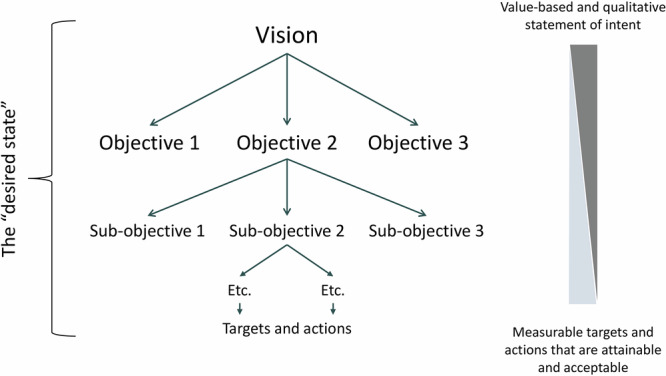
Generic objectives hierarchy representing a co-constructed and credible outcome, the desired state, which itself incorporates societal values, management pragmatism and scientific rigor

The objectives hierarchy explicitly linked societal values with pragmatic targets and actions, achievable through rigorous management, monitoring and reporting processes. A high-level statement of strategic intent is disaggregated into a hierarchical series of increasingly finer scale qualitative objectives, with increasing focus and achievability, mostly ending in quantitative, operational targets or actions (Fig. 2). Hence, in the hierarchy of objectives, an objective becomes a target or action when it reaches a level of specificity that is “achievable, testable, and auditable…with specific spatial, temporal and confidence limits” (Rogers and Biggs 1999, page 443). Note that these endpoints or targets were referred to as ‘goals’ in early SAM publications, but changed here to ‘targets’ in line with contemporary use of SAM and adaptive management more broadly. Targets are meant to address both governance (managing institutional structures and processes) and on-the-ground management (for ecosystems or social-ecological systems) endpoints.

Management targets and actions were supported by decision thresholds, termed ‘thresholds of potential concern’ (TPCs). TPCs were typically expressed as acceptable upper and lower levels, representing natural spatial and temporal variability in a particular ecological indicator. These were defined based on available information, drawing on the collective wisdom of researchers, managers and other local stakeholders. Their development and use were integrated into management and research programs, providing a focus for action and/or further investigation. The achievement of TPCs was monitored, evaluated, audited and revised (Rogers and Biggs 1999), informed by feedback from monitoring, directed research and observations (Kevin Rogers, personal communication, July 2023). Objectives hierarchies were operationalized through a ‘Goal Maintenance System’, allowing implementation, auditing and revision over time backed up by effective documentation to promote institutional memory (Rogers and Bestbier 1997).

### Initial Development (1997–2011)

SAM’s development for river management challenges happened when KNP, the oldest (established in 1926) and largest (about two million ha) of South Africa’s 20 national parks, needed a new management approach. This was necessitated by developments in ecosystem science as well as wide-ranging changes in environmental and socio-political contexts (Venter et al. 2008; Freitag et al. 2014; Carruthers 2017). In the 1990s, new insights in ecosystem science (e.g. Pickett et al. 1997; Levin 1999; Holling 2001) embraced complexity and heterogeneity, shifting from managing for an ‘ideal’ or stable state to dynamic states. Before, KNP used command-and-control (see Holling and Meffe, 1996) interventions to manage to a perceived stable state, including building of dams to water wildlife, culling of either predators or prey species to achieve an ‘ideal balance’, and using firebreaks and regular burning to control fire regime (Carruthers 2008). Acknowledging inherent uncertainty in complex adaptive systems required learning to be “maximized by using decisions, management actions and unexpected surprises…as experiments” (Venter et al. 2008, page 180). Research needed to be a partner to management, reflecting the credo of adaptive management (Walters 1986).

Furthermore, there was better appreciation of the effects of external (outside park) social-ecological influences on conserving biodiversity in KNP. Internal management issues such as fencing, wildlife population control, water-for-wildlife provision and fire policy were dwarfed by “massive, ‘external’ onslaughts” (Venter et al. 2008, page 181). These included natural ecosystem dynamics (e.g. animal population dynamics beyond the borders), threats (emergent diseases, invasive alien biota and a freshwater crisis originating upstream of KNP) and social pressures (vociferous and influential animal rights groups, indigenous land claims). Such issues required active engagement of relevant stakeholders, contrasting a previous lack of public consultation on management (Venter et al. 2008). Democratic change in South Africa in 1994 also drove principles of cooperative governance and public participation in decision-making (Freitag et al. 2014).

Thus, in 1997, KNP scientists and managers together with some external scientists and stakeholders used SAM to develop an objectives hierarchy for the park, involving various workshops and deliberations over nine months (Rogers and Bestbier 1997). Ratified at public meetings, the objectives hierarchy became a foundation for the first publicly mandated management plan for a national park in South Africa (Freitag et al. 2014). The process of developing an objectives hierarchy was later more aptly referred to as ‘adaptive planning’ (e.g., Roux and Foxcroft 2011), described most fully by Rogers and Luton (2011).

**Fig. 3:**
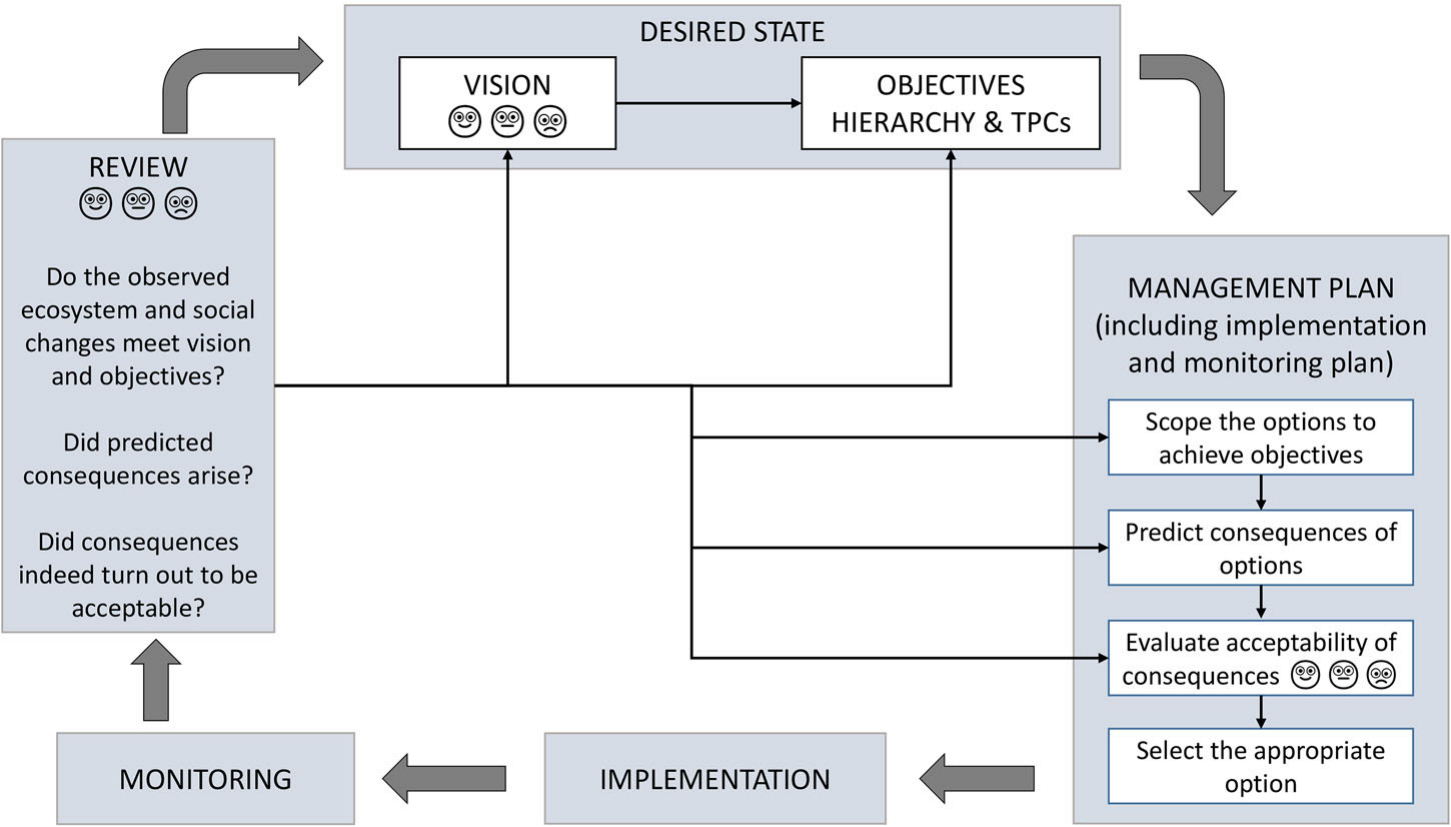
The early SAM process (after Rogers and Sherwill 2008), with thick gray arrows indicating sequential steps and thin black arrows feedbacks, with face emojis indicating where stakeholder involvement was deemed crucial

SAM’s development was fostered by South Africa’s National Environmental Management: Protected Areas Act (No. 57 of 2003; enacted in 2004), legally requiring development of management plans for all protected areas. Thus, SANParks (agency responsible for managing national parks in South Africa) adopted the SAM framework (Fig. 3; Rogers and Sherwill 2008) for management planning of all national parks. The SAM adaptive planning process gained credibility because it drove dialog, strengthened relationships and built trust among disparate stakeholders. This was particularly reflected in a high-profile and contentious elephant management debate (Biggs et al. 2008), where the transparency of SAM provided a legitimate outcome, even when values and objectives of all stakeholders were not fully reconciled.

During its initial development (1997–2011), SAM was predominantly implemented in river management (e.g. Rogers 1998; 2006; Pollard and Du Toit 2007; McLoughlin et al. 2011; Pollard et al. 2011), but also some invasive species management (Foxcroft 2004; 2009; Foxcroft and McGeoch 2011), elephant management (Biggs et al. 2008), fire management (Van Wilgen et al. 2011) and social dimensions of conservation (Scheepers et al. 2011; Swemmer and Taljaard 2011). Links between science and management were emphasized (Biggs and Rogers 2003), including through the co-development of TPCs (Biggs et al. 2011a; Kingsford et al. 2011) and purposeful co-learning to ensure the uptake of science in decision-making (Biggs et al. 2011b; Stirzaker et al. 2011).

By 2011, SANParks’ experience of SAM had comprehensively shifted its management, from mostly reactive and tactical responses to immediate issues, to a vision-oriented strategic approach driven by forward planning, evidence-based decision-making and learning that involved stakeholders (Freitag et al. 2014). This development and progress was captured in an 18-article feature of the journal *Koedoe* (Roux and Foxcroft 2011). While KNP was the flagship for the approach (Freitag et al. 2014), SAM became formally institutionalized throughout SANParks for planning and implementing park management plans (Rogers and Sherwill 2008).

Application of SAM extended beyond SANParks to water resource management in South Africa (Rogers et al. 2000a; McLoughlin et al. 2011; Rogers and Luton 2011) and generalized river conservation in protected areas, with the latter exemplified by case studies in Australia and South Africa (Kingsford et al. 2011). Of the 37 publications in the SAM database covering the initial development phase (1997–2011), all but two first authors were South African affiliated (94.6%), with all but three focused on a SANParks-related application.

### Geographic Spread and Further Development (2012–2023)

Thirty-seven relevant publications track the spread and further development and of SAM (2012–2023). It was predominantly applied to river and wetland management (Saintilan and Imgraben 2012; Rogers et al. 2013; Baumgartner et al. 2014; Parsons et al. 2016; Kingsford et al. 2017; Conallin et al. 2018a, 2018b; McLoughlin et al. 2021) as well as integrated water resource management (Palmer et al. 2018). This included groundwater (Bouchet et al. 2019) and water services (Frame et al. 2018). A standout contribution in the aquatic context was an IUCN report on “guidelines for effective conservation of freshwater ecosystems in and around protected areas of the world” (Kingsford and Biggs 2012). Other SAM applications included resource use and benefit sharing (Swemmer et al. 2015), learning (McLoughlin and Thoms 2015; Rosenberg et al. 2018; McLoughlin et al. 2020; Roux et al. 2022), fire management (Van Wilgen et al. 2022; Strydom et al. 2023), tourism (Biggs et al. 2014) and megaherbivores (Smit et al. 2020).

SAM’s landscape-scale management applications also increased, including rural landscape restoration and livelihoods (Fabricius et al. 2016; Cockburn et al. 2018, Palmer et al. 2023), river catchments (Palmer et al. 2018), agricultural landscapes (Kreiling et al. 2018), restoration of desert ecosystems (Kingsford et al. 2020), woodland restoration and livelihoods (Moyo et al. 2021) and national parks (Roux et al. 2022). Consequently, SAM became conceptually linked to adaptive governance (Novellie et al. 2016; Roux et al. 2023) and explicitly incorporated social-ecological systems thinking, sensu Berkes and Folke (1998) (Anthony and Swemmer 2015; Parsons et al. 2016; Cockburn et al. 2018; Conallin et al. 2018a; Bouchet et al. 2019; Palmer et al. 2023). Also, Gillson et al. (2018) outlined synergies between adaptive management (modeled on SAM) and evidence-based approaches in conservation.

While the initial development of SAM primarily focused on the adaptive planning process (i.e., for setting a desired state) and less on implementation, the extended development phase gave more explicit attention to how hierarchies of objectives were operationalized (e.g. Conallin et al. 2018a; Palmer et al. 2018). Further, during SAM’s early development, negotiating for a consensus view of the desired state was seen as critical (Rogers and Bestbier 1997; Rogers et al. 2000b). In recent years, more pluralistic approaches (see Cockburn et al. 2019) have found traction, emphasizing inclusive dialog and social learning while promoting shared understanding and co-producing knowledge for a common purpose (Palmer et al. 2023; Roux et al. 2021, 2022, 2023). Hierarchies of objectives also increasingly included governance and process objectives (e.g. Kingsford et al. 2020; Smit et al. 2020; Roux et al. 2021), in addition to ecosystem management objectives that dominated during the initial development phase.

During the 2012–2023 phase, use of SAM spread geographically beyond South Africa (also see Freitag et al. 2014), evidenced by 21 first-author affiliations from South Africa (56%), 15 from Australia and one from Hungary. Apart from a clear Australian development, South African publications spread to rural landscapes, primarily in the Eastern Cape Province of South Africa (Fig. 1).

## Present-day SAM

In this section we characterize present-day SAM, drawing on insights gained from three semi-autonomous and parallel streams of development: SANParks, Australia and Eastern Cape (Fig. 1). First, we briefly describe the contexts of the Australian and Eastern Cape streams, adding to the already described SANParks stream. Second, we use our combined experiences from all three streams, including their respective strengths and weaknesses, to characterize present-day SAM. Third, we reflect briefly on how SAM relates to some other adaptive management forms and approaches, prominent in the literature.

### Context of Australian and Eastern Cape streams

#### Australia

Application of SAM in Australia has predominantly been at the landscape scale, focusing on freshwater and terrestrial ecosystems (Kingsford et al. 2011, Kingsford et al. 2020, 2021). Most applications are published but some recent initiatives are also underway. These unpublished approaches involve a range of government and non-government organizations, indigenous groups and universities, across central and south-eastern Australia.

Application of SAM began primarily on management of environmental flows in the Murray-Darling Basin, among Australia’s largest and more developed river systems (Kingsford 2000). The Australian Government dedicated more than 13 AUD billion to buy back allocated water from the irrigation industry and by improving water use efficiency, returning it as environmental flows (Swirepik et al. 2016). This water for the environment is held as licenses, allocated each year as a share of storages in dams, similar to the management of irrigation licenses (Steinfeld et al. 2020). There was a focus on identifying a high-level hierarchy of objectives for the Ramsar-listed Macquarie Marshes (Kingsford et al. 2011), linked to the management of environmental flows. However, management of flows to the environment continues to largely reflect institutional practices, informed by legislation and policy, without clear explicit objectives to guide assessment of effectiveness and improve learning (McLoughlin et al. 2024). SAM was also independently used to guide stakeholder engagement and decision-making for environmental flow management in the Edward-Wakool system of the River Murray, identifying the importance of co-design (Conallin et al. 2018a). It was also used to build a vision and high level objectives hierarchy for the Coongie-Malkumba Lakes, focused on Australia’s largest Ramsar site in South Australia, with multiple uses including grazing, oil and gas, strong traditional owner engagement, and a national park (Kingsford et al. 2021). It also underpins restoration management of the Gayini Wetlands on the Lower Murrumbidgee floodplain ( ~ 80,000 ha), under the leadership of the Nari Nari Tribal Council, supported by conservation and science (Woods et al. 2022).

There are also two examples of SAM implementation in terrestrial ecosystems. First, SAM underpins management and investment in the Wild Deserts Project in Sturt National Park. This reintroduction or rewilding project is focused on reintroducing seven locally extinct species, a partnership involving Ecological Horizons and the New South Wales National Parks and Wildlife Service. Practitioners responsible for the management, supported by science, are implementing SAM over 35,000 ha of Sturt National Park (Kingsford et al. 2020). The SAM plan incorporates social dimensions, including partnerships, outreach and internal capacity to deliver on complex objectives. The SAM plan continues to evolve, incorporating more operational functionality in relation to transparency, planning, communication, implementation, and reporting. Second, SAM was developed for the restoration management of UNSW Sydney field research station at Fowlers Gap in the arid zone (39,000 ha). In 2021, UNSW removed sheep (*Ovis aries*) and focused on restoration. A major program began with erection of fenced exclosures to test the effects of different grazing species, including European rabbits (*Oryctolagus cuniculus*), macropods (e.g. *Macropus rufus*) and feral goats (*Capra hircus*) and foxes (*Vulpes vulpes*) to protect native species. A SAM plan was developed, incorporating restoration ecology in a vision and a hierarchy of objectives, reflecting objectives of education, research and outreach. The detailed hierarchy of objectives focused on actions to remove sheep, reduce grazing pressure by removing artificial waters.

#### Eastern cape and beyond

Researchers at Rhodes University, through the Institute for Water Research, the Department of Environmental Science and the Environmental Learning Research Centre, applied SAM to landscapes and river catchments in the Eastern Cape, South Africa. Through the place-based implementation of SAM in the Eastern Cape led by researchers at Rhodes University, this stream has added to the intellectual project of SAM through practical application and published research (Fig. 1). The Eastern Cape stream drew on experiences from the KNP, through collaborative research on a basin-scale water resource management project (Palmer et al. 2018; Palmer and Munnik, 2018) and a multi-national initiative on reducing vulnerability to climate change (AWARD, 2019). A flagship SAM initiative was the ‘Tsitsa Project’ (2014–2022), in the Tsitsa Catchment, a sub-catchment of the Umzimvubu River in the Eastern Cape (Cockburn et al. 2018; Powell et al. 2018; Tsitsa Project et al. 2021a, Palmer et al. 2022). The Tsitsa Project emerged because of siltation concerns in two proposed large dams (Powell et al. 2018), with primary funding by the South African government through its Department of Environment, Forestry and Fisheries (DFFE). The DFFE committed to maintaining healthy upstream ecosystems (‘ecological infrastructure’; Cumming et al. 2017) to prolong the lifespan of the proposed dams and support sustainable land-based livelihoods for local people. DFFE invested in a multi-year partnership with researchers from Rhodes University, and other partner universities, to implement science-based integrated landscape management and restoration (Powell et al. 2018), enabled by a participatory governance process (Palmer et al. 2022) and embedded in a transdisciplinary research framework (Cockburn et al. 2018).

The Tsitsa Catchment was framed as a complex social-ecological system, adopting a SAM approach to natural resource management (Fabricius et al. 2016; Biggs et al. 2019), implementing SAM’s three sub-processes: adaptive planning, adaptive implementation and adaptive evaluation (Roux and Foxcroft 2011). The Tsitsa Project drew on lessons learnt by AWARD (2019), particularly related to Participatory Monitoring, Evaluation, Reflection and Learning in its application of SAM (Tsitsa Project et al. 2021b). This required a knowledge management and mediation officer and a platform to capitalize on and utilize diverse information and knowledge. A ‘desired endpoint’ or vision was co-developed with stakeholders, including local catchment residents, through adaptive planning (Cockburn et al. 2018; Palmer et al. 2022). Based on the vision, an objectives hierarchy was developed, primarily involving managers and scientists, revisited and refined during the project. SAM was an on-going process, happening at different levels in the project at different times. A key learning in the Tsitsa Project was working respectfully with a diversity of stakeholders (Rosenberg et al. 2023), and recognition for the importance of individual and collective agency for effective governance and transformation (Weaver et al. 2023; Palmer et al. 2022).

The Tsitsa Project’s exploration and expansion of SAM are informing other initiatives, including newly emerging ‘Adaptive Systemic Approach’, which is now being trialed in seven other African countries (Palmer et al. 2023; Palmer and Tanner, 2024). This, together with the production of academic papers reflecting on SAM implementation, demonstrates the significance of the Eastern Cape stream in the further conceptual and practical development and spread of SAM. The ‘Eastern Cape and beyond’ stream of SAM is influenced by interdisciplinary researchers at Rhodes University, drawing on theory and methodology from the social sciences, in particular, to inform research and reflexive, adaptive implementation of SAM (e.g. Cockburn et al. 2018; Palmer and Tannerlee 2024). This has been achieved through engaged research in fields such as governance (e.g. Ralekhetla 2018; Fry et al., 2023), education (e.g. Mtati 2020; Lotz-Sisitka et al. 2024), evaluation (e.g. Human, 2020; Rosenberg and Kotschy 2020), and other social science expertise.

### Strengths, Weaknesses and Key Characteristics

**Fig. 4:**
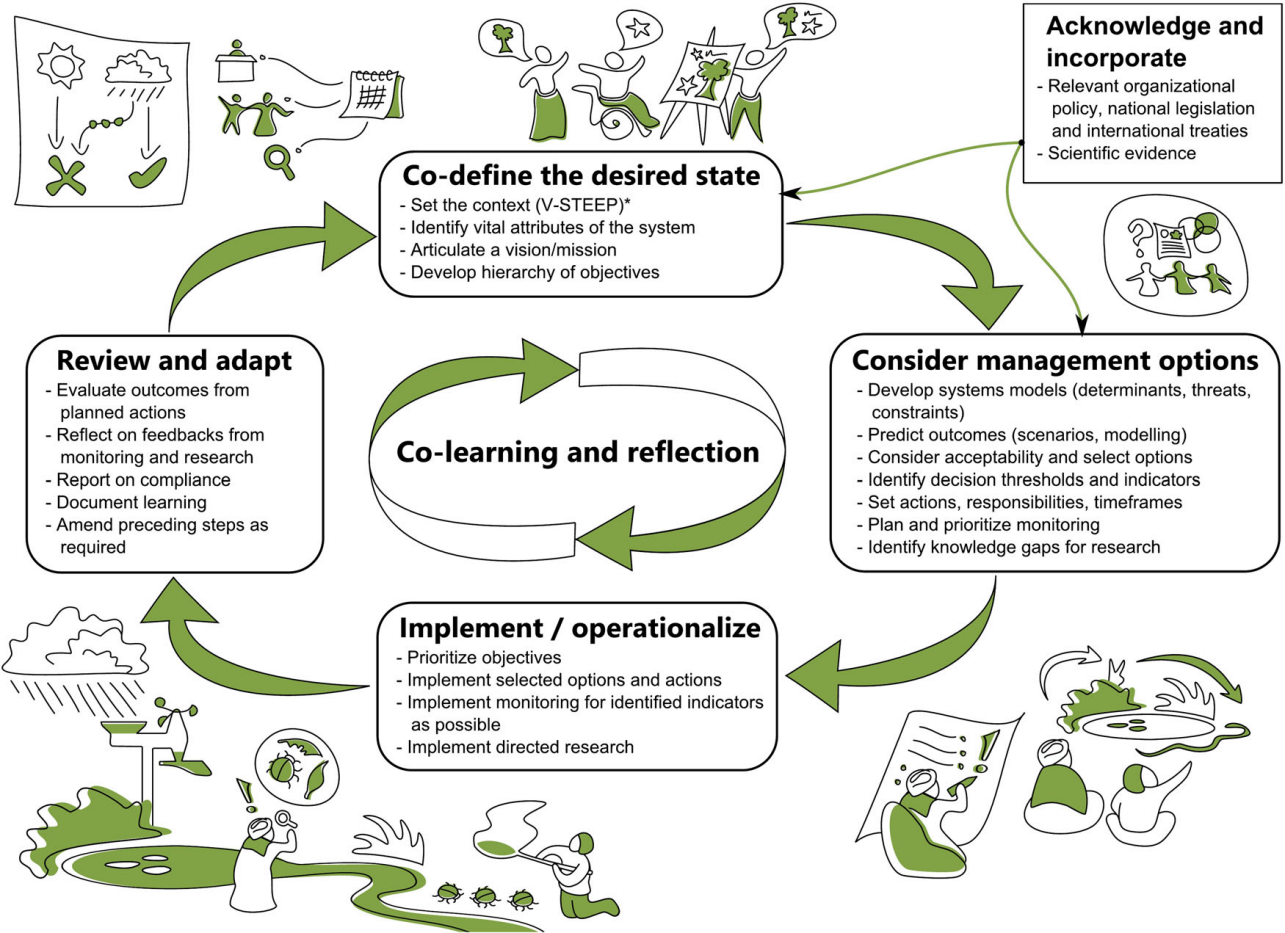
Generalized process diagram for present-day Strategic Adaptive Management (SAM), showing four semi-sequential steps forming a reinforcing loop with cross-cutting co-learning and reflection in the center. V-STEEP in the co-define step serves as a reminder that the process starts with agreeing on Values and deliberating how these might be achieved within the constraints of changing Social, Technological, Economic, Environmental and Political contexts

We identified strengths and weaknesses across the three streams of SAM (Table 2). Drawing on these and our collective experience, we developed a generalized SAM framework (Fig. 4) that reflects current practice across all streams. Next we use the framework’s four main steps (co-define the desired state, consider management options, implement / operationalize, and review and adapt) together with its cross-cutting process of co-learning and reflection, to describe the key characteristics of present-day SAM practice.

**Table 2:** Strengths (**S**) and Weaknesses (**W**) of different steps and elements identified within the three parallel streams of Strategic Adaptive Management (SAM)

Step	Element	SANParks	Australia	Eastern Cape (Tsitsa Project)
Co-define the desired state	Vision and high-level objectives (co-produced)	**S**-Taking care to co-produce the vision and high-level objectives promoted trust among stakeholders (Roux et al. 2021), with the products serving as a people’s theory of change (Roux et al. 2023).	**S**-Each project area had inclusive dialog to develop a shared vision, crafted by stakeholders. Sometimes stakeholder engagement was limited because of project scope (e.g. Kingsford et al. 2020a).	**S**-The vision was co-produced, iteratively, with two different sets of stakeholders as the partnership grew and diversified.
	Focus on making social-ecological values of stakeholders explicit	**S**-A rigorous process for making these values explicit promoted multi-stakeholder transparency and trust, and helped with prioritization of management.	**S**-Provided a rigorous process for identifying these values for prioritizing management.	**S**-Provided a legitimate and structured way of engaging diverse social-ecological values and hearing different voices.
	Inclusive stakeholder engagement	**S**-Supported by national legislation and SANParks policy, genuine efforts were made to achieve inclusive dialog among relevant stakeholders. **W**-Has proven difficult to sustain meaningful engagement over long periods because of fatigue and resourcing constraints (Roux et al. 2021).	**S**-Stakeholder engagement is critical **W**-Can be challenging to define stakeholders (e.g. Kingsford et al. 2020b) and resource engagement over time.	**S**-Caring, careful and respectful relationship- and trust-building informed stakeholder engagement and were essential for understanding the complex sustainability issues (Rosenberg et al. 2023); specific skills and resources and commitment to inclusivity were also important (Palmer et al. 2022).
	Explicit development of key social, ecological and governance goals, identified as objectives	**S**-This promoted systemic thinking about complex social-ecological systems and enabled bridging from single-authority management to collaborative governance where appropriate (Roux et al. 2023).	**S**-This has allowed improved specification of social as well as ecological objectives, particularly in relation to governance, stakeholders, outreach and resourcing.	**S**-SAM and the broader transdisciplinary approach of the project enabled a focus beyond just the usual natural resource management (ecological) objectives to include social, governance and also integrated ‘social-ecological’ objectives.
	Hierarchy of objectives (co-produced)	**S**-Were co-produced by managers and scientists, with involvement of other stakeholders on key objectives; linked high-level aspirational objectives for systems and issues to pragmatic management actions.	**S**-Specific objectives define the key actions required to achieve the desired state and its high level objectives within the stakeholder defined group.	**S**-Developing the hierarchy of objectives through diverse researchers together in a transdisciplinary approach. **W**-Never fully implemented due to unexpected funding cuts and shifts in high-level political priorities.
Consider management optionsImplement / operationalize actions, monitoring and research	Conceptual models and scenarios	**S**-Systems models (mostly conceptual) were used as ‘boundary objects’ to facilitate sense-making and coherence across diverse perspectives. **W**-Due to time constraints, conceptual models were only used for select objectives and scenarios rarely used.	**S**-Useful to identify different mental models, even if not explicitly defined as a conceptual model which may confuse. For technical quantification, an explicit conceptual model reduces ambiguity on drivers and response variables to be measured for scenarios. **W**-Engagement processes were long and did not always identify scenarios or conceptual models explicitly.	**S**-Systems models were used in the early stages (primarily with scientists and some managers, not local community residents); ‘learning words’ workshops were facilitated with local language speakers to co-develop a systems understanding (Palmer et al. 2022). **W**-Typical systems models can be alien to people who are not used to them.
	Decision thresholds	**W**-Formulaic development of thresholds of potential concern (TPCs) for all issues and their implementation was found overwhelming and avoided.	**S**-Decision thresholds identified as needed and used in implementation. **W**-Some thresholds needed more data, providing a focus for resourcing; needed to be careful about not overwhelming decision space with data.	**W**-Detailed refinement of decision thresholds (TPCs) was considered infeasible due to the complexity of the initiative and limited resources for fine-scale implementation and monitoring.
	Action plans	**S**-Actions, responsibilities and time-frames were made explicit in park management plans, which were gazetted by the national government. **W**-Including operational detail in a statutory plan impaired flexibility of SAM (Novellie et al. 2016).	**S**-Clear actions, sometimes detailed as action plans with accountabilities are essential. These were primarily identified as the lowest level of the objectives hierarchy.	**W**-The objectives hierarchy was refined down to the level of actions, but implementation and monitoring at this level of detail was constrained; additional actions were at times determined in a more ad-hoc manner, based on opportunities for collaboration or emergent resources (funding) or issues.
	Monitoring and research programs	**W**-Due to time constraints, designing monitoring programs and identifying research gaps were rarely undertaken during this step and typically deferred for later development.	**S**-It is important to have clear protocols for monitoring and research, which could be expressed as objectives. Research targets gaps in knowledge. **W**-Sometimes poorly developed and not well linked to values and vision.	**S**-The development of Participatory Monitoring, Evaluation, Reflection and Learning (PMERL), and resource allocation to monitoring and research from the start, as well as early implementation, was a key strength of the initiative; doing this in a transdisciplinary way by involving diverse stakeholders built capacity and cohesion among participants (Cockburn et al. 2018).
	Resourcing	**S**-An institutional commitment to SAM made resourcing central to management planning. **W**-Resourcing (human and financial) was not sufficient for implementing, monitoring and researching all objectives and indicators.	**S**-Investment in required resources is essential. Sometimes ‘research’ is interpreted as ‘blue sky’ rather than essential for monitoring and effective management. Monitoring and research are included as actions or action plans, sometimes requiring funding. **W**-Most implementation has occurred outside government agencies in control of resourcing. Without internal champions there is limited continuity, essential for implementation.	**W**-Funding and shifts in high level priorities meant that the Tsitsa Project vision, objectives and ambitious monitoring program were not fully implemented.
	Scientific focus	**S**-Having explicit objectives and identified actions enabled effective and targeted science. **S**-Embedded research scientists can respond to real management needs and serve as conduits between park management and academic researchers.	**S**-Specification of specific objectives provides the key knowledge requirements for effective and targeted science. Important to ensure well designed. Challenging to manage but important to have both constancy for repeatability and tracking change through time but also flexibility for particular unplanned issues. **W**-Rigorous design is not always adequately considered important.	**S**-Drawing on diverse scientific disciplines (including social sciences such as education, development studies, etc.) to conduct research in the catchment ensured that scientific objectives were aligned to the complexities of the social-ecological system in focus.
	Monitoring	**S**-Much monitoring is being done. **W**-It has proved difficult to modify or discontinue existing monitoring programs to align with latest management objectives (Van Wilgen and Biggs 2011; Roux et al. 2022). Also, resources are insufficient to enable monitoring of, and reporting on, effectiveness of all objectives and their actions.	**S**-For Wild Deserts, there is rigorous monitoring of effectiveness for decision-making (Kingsford et al. 2020b). **W**-Other projects have limited follow through or prioritization of monitoring.	**S**-An ambitious monitoring process was put in place and started through a PMERL system. **W**-Unfortunately, it failed to reach its objective in the long term due to resource and institutional constraints.
Review and adapt (including evaluation for compliance / auditing)	Transparency	**S**-This was achieved through clear articulation of objectives and associated management actions, and further enhanced through evaluation/ reflection and reporting, which is essential for a public-sector agency.	**S**-There is clear articulation of objectives for management actions, supported by transparent reporting. **W**-This can be confronting for government and non-government partners where there are failures. Important to admit and learn from failures.	**S-**Clear articulation of a shared vision, objectives, actions, and indicators, as well as a monitoring system, set the foundations for transparency. **W**-However, this was seen as a ‘parallel’ system outside formal government processes and perceived as a threat by some due to its transparent nature and openness to acknowledge failure.
	Reporting	**S**-Quarterly and annual reporting on outputs are being done; the embedded science function published some lessons learnt in scientific literature (e.g. Smit et al. 2017). **W**-There seemed to be little if any feedback from higher authorities on reports being produced; attention to internal documentation and sharing of lessons is sub-optimal.	**S**-Reporting is essential for funders and outreach on results of investments. **W**-Sometimes over reporting can be a challenge. Effectiveness primarily depends on responsibilities and commitments of key organizations. Processes often inadequately link to outputs on outcomes for reporting. Important to also link reporting to decision-making.	**S**-The leading funder had stringent reporting requirements (quarterly and annual), which aligned well with SAM and PMERL approach and enabled feedbacks and adaptation along the way. **W**-Questions remain as to who read reports and acted on them other than key project leaders (i.e. did government partners read and engage with reports other than for funding compliance, and did the reports inform decision-making and implementation beyond the core project team?).
	Managing multiple hierarchical objectives	**S**-Helped to make explicit the complexity of the task including tensions between sometimes conflicting objectives. **W**-Too many objectives linked to multiple actions, especially when subject to auditing, can become overwhelming.	**S**-For Wild Deserts (Kingsford et al. 2020), level of detail required for recording of transparency of management achievements. **W**-Too many hierarchical objectives may be practically difficult to manage for auditing and ensuring sufficient utility without onerous administrative burden.	**S**-Attempts were made to engage with the complex objectives hierarchy through regular PMERL reports. **W**-The momentum was difficult to maintain and it was not possible to include diverse stakeholders in this process as it was somewhat alienating (“overly scientific”) and time-consuming.
	Operationalising SAM through annual auditing and tracking of objectives	**S**-Annual tracking tool assessments helped to keep operational efforts aligned with set objectives.	**S**-In Wild Deserts project (Kingsford et al. 2020b), there is an annual listing of SAM hierarchy of objectives, allowing ongoing objectives (including high level actions) to be ‘rolled’ into subsequent years and completed objectives recorded and archived. **W**-Identification of the manageable level of objective specification to reduce the administration burden is essential.	**S-**For core project leaders and champions annual auditing and tracking worked well in the early stages. **W**-It was difficult to maintain momentum and ensure inclusivity in this process, and to embed this into standardized government reporting and decision-making processes (it remained) somewhat of an ‘artifact’ of the researcher-led project.
	Reflexive learning	**S**-Institutionalized science-management forums promote co-reflection and co-learning between science and management functions. **W**-Compliance-style evaluation stifles deeper reflection; external stakeholders are mostly absent during evaluative learning.	**S** - Important in some projects to use SAM as the main basis for decision-making in complex projects. Constant learning is inevitable with new data and understanding. **W**-Difficult to include stakeholders in learning given inadequate continuity and maintenance over long periods.	**S**-PMERL added depth and contextual detail to the adaptive evaluation step in SAM by developing indicators for the objectives hierarchy in a participatory process, along with monitoring tools, protocols, resources and reflective learning processes; these were designed to include and benefit catchment residents as much as possible.
Co-learning and reflection (cross-cutting step)	Reflection and learning culture	**S**-Through SAM, learning is institutionalized. **W**-Statutory status of park management plans drove an emphasis on achievable and auditable actions at the cost of learning opportunities, hampering the learning potential of the SAM process.	**S**-Learning is implicit throughout the different projects and an essential part of collaboration. **W**-Learning is not well understood or captured.	**S**-SAM enabled development of learning culture; partnering with education researchers helped to strengthen research and implementation around learning aspects. Implementing SAM and PMERL requires specialized skills and knowledge, across the domains of technical, relational and transformational competences (Rosenberg and Kotschy, 2020).
	Ongoing learning	**S**-Ongoing learning promoted through review of progress in the context of new information resulting from monitoring, research, observations, changing contexts and emerging issues.	**S**-Highly dependent on resourcing and organizational commitment in relation to longevity of SAM. With resourcing from government and non-government agencies, there is significant opportunity for ongoing learning, particularly with new information and understanding.	**S**-SAM and the transdisciplinary orientation of the project enabled, through PMERL, much learning, and early evidence of a ‘learning culture’ until the premature end of the funded project. **W**-In the long-term, on-going learning will require sustained resources and effective coordination, which has not been possible in this project context.
	Learning throughout SAM cycle	**S**-Acknowledged that each step of SAM enables different types of learning that in turn help to inform each subsequent step (Roux et al. 2022).	**S**-There is significant learning at every step as stakeholders and managers understand the explicit dimensions of problems. Inevitably increased understanding promotes further learning, coming from monitoring of actions and successful or unsuccessful achievement of outcomes. **W**-Generally poor understanding of the learning process and its effectiveness and importance.	**S**-Implementing the PMERL system ensured learning was focused on throughout, though this needed specific allocation of resources and key skills in the coordination team.
	Design and facilitation of inclusive learning spaces	**S**-Several institutionalised learning spaces were resourced to sustain co-learning among stakeholders. **W**-It was challenging to design for and achieve inclusiveness in a particular learning space when stakeholders have diverse backgrounds and expectations.	**S**-Opportunities for learning but requires constructive and ongoing engagement of stakeholders and collaborators, including those involved in the project. **W**-Important to explicitly identify processes and spaces for this to occur, including target audiences.	**W**-Learning spaces initially favored scientists and managers in terms of location, language and facilitation approach. **S**-Later, a more inclusive approach was taken to the design and facilitation of learning (Palmer et al. 2022; Weaver et al. 2023).

#### Co-define a desired state

Across all streams, this step had many strengths (Table 2). It helped focus the purpose of management around maintenance of social and ecological values, with diverse stakeholders operating as both a ‘sounding board’ and collaborators. This requires inclusivity, care and a commitment to meaningful relationship-building. Following a structured process, stakeholders agree on social and ecological values, including identified vital attributes or special features of the system, contextualized within dynamic social, technological, economic, environmental and political settings (Fig. 4; Rogers and Luton 2011; Kingsford and Biggs 2012; Palmer et al. 2018; Roux et al. 2021).

This adaptive planning process sets the tone and serves as springboard for later steps. It establishes an ethos of co-learning among stakeholders, entrenches a social-ecological systems perspective, encapsulates a value-based and co-defined vision to guide future decision-making, and aids in developing trust and a spirit of collaboration in often contentious natural resource management settings, involving diverse stakeholders (Cockburn et al. 2018). Importantly, diverse voices and perspectives need to be valued and incorporated, ensuring that stakeholder engagement is truly inclusive and even transformative (Palmer et al. 2022; Weaver et al. 2023).

Depending on circumstances, deliberations may range from one to many meetings, with up to hundreds of stakeholders co-producing a trusted vision and high-level objectives (Roux et al. 2021; Tsitsa Project et al. 2021a). These inform a hierarchy of objectives with increasing specificity, from high-level aspirational statements of intent, eventually linked to pragmatic management actions (see next step). This planning is resource intensive, proving challenging to sustain enduring engagement with stakeholders (SANParks and Australia streams), including implementation of the hierarchy of objectives without institutional endorsement and long-term funding (Eastern Cape).

#### Consider management options

The hierarchy of objectives, with systems models and scenarios, forms the basis for identifying management options. These are further informed by relevant legislation, scientific evidence and resource realities (Fig. 4). Selected options are linked to responsibilities, resources and timeframes, specifying specific actions, endpoints, indicators and decision thresholds, where relevant. The outputs of this as well as the previous step are typically captured in a management plan, which is sometimes accompanied by a separate monitoring program(s) or framework(s) (Tsitsa Project et al. 2021b).

Experiences on utility of models and scenarios had strengths and weaknesses (Table 2), from using quantitative models to reduce ambiguity on drivers and response variables (Australia) to stakeholders finding conceptual models confusing (Australia and Eastern Cape). Overwhelming decision thresholds for multiple objectives was intractable across all three streams; with operational detail required in action plans difficult to realize (Eastern Cape). Contrastingly, SANParks had too much detail in statutory plans, impairing flexibility. Linking monitoring and research directly to objectives was weak in the SANParks and Australian streams, while strong emphasis on this element in the Eastern Cape stream was a strength.

This step could be improved by careful prioritization of objectives for which to develop decision thresholds, to avoid becoming overwhelmed in multi-objective contexts (e.g. SANParks). Decision thresholds (e.g. TPCs) provide managers with an endpoint to aim for, and scientists a testable hypothesis. Identifying and prioritizing monitoring and research is also important at this step, drawing on diverse disciplinary perspectives and knowledge forms (including local and indigenous knowledge). This also builds capacity for local stakeholders to ensure meaningful participation (Cockburn et al. 2018; Palmer et al. 2022; Roux et al. 2023), as exemplified by work done in the Eastern Cape stream on Participatory Monitoring, Evaluation, Reflection and Learning (Rosenberg and Kotschy, 2020).

#### Implement/operationalize

The SAM management plan that results from the previous two steps guides day-to-day implementation. However, with uncertainty and surprise across multiple and sometimes conflicting objectives, making progress often feels like navigating a ‘bumpy terrain’, characterized by many tensions (Cockburn et al. 2018). In reality, the full hierarchy of objectives with monitoring and research are usually only partially implemented. Nevertheless, SAM provides transparency in decision making and drives ongoing learning and improved practice, even with far from perfect implementation.

Institutionalization of SAM was easier for SANParks, where embedded champions (from across management and science functions) and statutory endorsement supported long-term continuity and investment. This was more difficult for Australian and especially Eastern Cape initiatives, where external champions (university scientists) relied on grant funding with project time frames. SAM’s transparency enabled science to target key knowledge needs, aligning to place-based complexities. Embedded scientists in SANParks served as conduits between park management and external scientific knowledge. Although much monitoring was done across all three streams, this remained constrained by available resources (SANParks and Eastern Cape).

Structured approaches, such as SAM, are essential for effective conservation management. Opportunities for increasing knowledge of the effectiveness, transparency and rigor of SAM among government and non-government practitioners will increase potential uptake and continually improve implementation.

#### Review and adapt

This step has been categorized as ‘review’ (Rogers and Sherwill 2008; Fig. 3), ‘evaluation and learning’ (Kingsford and Biggs 2012; Kingsford et al. 2017; Roux et al. 2022) and ‘adaptive evaluation’ (Novellie et al. 2016). The name change over time reflects increasing emphasis of assessment-type evaluation (i.e. an immediate evaluation of performance) over deep review (i.e. reflection on and learning about action outcomes to inform adaptation when necessary). This has been attributed to an ever-growing compliance and accountability culture (Biggs et al. 2011b; Roux et al. 2022). We use ‘review and adapt’ (Fig. 4; Table 2), to emphasize elicitation and capture of lessons to improve future actions, together with evaluation and reporting for compliance and accountability of effectiveness.

Across all streams, frequent review to adapt helped make sure that a SAM plan tracked the desired state or vision. This happened through formal and informal ‘evaluative moments’, including tracking-tool assessments, stakeholder fora, co-reflection sessions, collaborative monitoring (e.g. through citizen science, see Rosenberg et al. 2023) and participatory research or field visits. Formal reviews in SAM were a strength for public-sector SANParks, but caused potential discomfort and even threatened power because of potential failure (Australia and Eastern Cape). Reporting on review findings was firmly entrenched across all streams, but sharing the lessons effectively with key audiences remained challenging. SANParks found that reviews promoted co-learning between science and management functions (strength) but compliance-oriented evaluation stifled reflection. Involvement of diverse stakeholders in review and reporting was challenging and minimal for SANParks and Australia, contrasting the Eastern Cape, working with catchment residents (Table 2; Tsitsa Project et al. 2021b).

#### Co-learning and reflection

Learning was central to and occurred throughout the SAM cycle across all streams (Table 2). This contrasts with depictions of learning in literature on earlier SAM (e.g. Fig. 3) and adaptive management generally (Westgate et al. 2013), as feedback from research, monitoring and evaluation. In reality, (a) valuable learning happens at every step of the process; (b) learning acquired during each step feeds into and enriches learning at the next step; and (c) each SAM cycle builds on the learning from previous cycle to systematically consolidate learning at all scales from individual to organization and stakeholder networks (Roux et al. 2022).

All streams had success but also experienced challenges with designing inclusive learning spaces (Table 2). Weaknesses experienced include that learning was stifled when statutory responsibilities drove actions focused on achievability rather than learning opportunities (SANParks); not always well understood or captured (Australia); and difficult to resource effectively with short-term project funding (Eastern Cape). Ultimately, effective co-learning builds capacity and enables transformative change, with many systemic benefits for participants, improving effectiveness of SAM in achieving management objectives (Cockburn et al. 2018; Weaver et al. 2023).

### SAM in Relation to Other Adaptive Management Approaches

Adaptive management has taken on many forms and interpretations (Rist et al. 2013a; Hasselman 2017) since first proposed more than four decades ago. While it is beyond the scope of this paper to review the different forms and approaches in any detail, we briefly reflect on how SAM relates to some of the prominent ones.

Two disparate paths have emerged in adaptive management literature. ‘Active’ adaptive management involves strong experimental design, aimed to efficiently separate competing hypotheses about effects of management actions. It can require substantial resources for planning, implementation and monitoring (Walters 1986; Walters and Holling 1990). ‘Passive’ adaptive management uses available information to develop hypotheses for a preferred management action, with monitoring of outcomes informing future adaptations (Gregory et al. 2006). This form lacks replication and controls (Van Wilgen and Biggs 2011), making it less powerful in identifying cause-and-effect relationships than experimentation. Both active and passive forms use formalized learning to inform adjustment of management actions (Rist et al. 2013a), distinguishing it from ‘trial and error’ management where there is no explicit and formalized process for ongoing learning.

SAM applications predominantly reflect passive adaptive management (Van Wilgen and Biggs 2011), with additional emphasis on devising directed research and incorporating its findings in decision making. Although experimentation is also used (e.g. Van Wilgen et al. 2011; Kingsford et al. 2020), an early decision was that relying on experimentation as a main source of learning would be too restrictive in largely uncontrollable social-ecological contexts such as river basins (Kevin Rogers, personal communication, November 2024).

A distinct branch of adaptive management is adaptive co-management (ACM; Armitage et al. 2009; 2010; Plummer et al. 2012; Hasselman 2017), described as the convergence of adaptive management and co-management (two or more actors sharing management responsibilities, Armitage et al. 2010). It centers on combining strengths of these two approaches, namely learning and linking (Plummer et al. 2012). ACM “empowers resource users and managers in experimentation, monitoring, deliberations and responsive management of local scale resources, supported by, and working with, various organizations at different levels” (Hasselman 2017: 37). It is similar to SAM, but has evolved and is implemented in different academic and practitioner communities and contexts (e.g. coastal and marine systems). Contrastingly, SAM is more commonly applied in terrestrial and freshwater systems. Both have goal-oriented management, learning, and multi-stakeholder collaboration with two notable differences. Firstly, ACM places more explicit emphasis on cross-level and cross-scale linkages (vertical and horizontal) and potential power asymmetries in multi-actor and multi-institutional partnerships managing social-ecological systems (Armitage et al. 2010). While SAM accommodates such cross-scale linkages and can enable effective conflict management, power considerations have not been addressed much in the SAM literature. Secondly, SAM provides a more fine-scale, step-wise and pragmatic operational model for management and monitoring, unlike the focus of ACM on collaboratively managing uncertainty and conflict through a more generic framework.

A further prominent approach in adaptive management literature is Open Standards for the Practice of Conservation, or simply Conservation Standards (Conservation Measures Partnership 2020). Focusing on conservation management and developed and supported by non-government organizations and government agencies, Conservation Standards has many different applications around the world (Schwartz et al. 2012; Núñez-Regueiro et al. 2020; Brown et al. 2022). It comprises five steps (Conservation Measures Partnership 2020): assess, plan, implement, analyze and adapt, and share. Conservation Standards and SAM are similar in several aspects, with both focusing on the entire process of conservation or environmental management; recognizing the importance of engaging stakeholders and learning at every step; and rigorously documenting a process of identifying and defining a vision, informed by an assessment of values and threats. From these flow relevant management actions that are implemented, analyzed and adapted (Conservation Standards) or reviewed and adapted (SAM). A key difference is that Conservation Standards has a well-developed approach to assessment and problem definition, requiring an important analytical step, primarily focused on assessment of threats and values. Contrastingly, SAM specifies the context (including current understanding), focused on values, drivers and threats, but inevitably constrained by social, technological, economic, environmental and political conditions (V-STEEP; Fig. 4). The conservation problem is considered broadly well understood, and defined by the context. Conservation Standards utilizes result chains to link goals to objectives and activities, while SAM employs the objectives hierarchy to link a vision through multi-level objectives to actions. SAM only uses the noun ‘objectives’ for simplicity to describe goals, targets, actions and activities, recognizing that higher level objectives are more value laden compared to actionable lower-level objectives (Fig. 2).

We argue that there is a place for different forms of, and approaches to, adaptive management, with the appropriate form being a function of management context and need, which may vary over time and space across the application landscape. At least some differences, e.g. in terminology, may simply reflect policy and other contexts of a particular time and place. Even though Conservation Standards, SAM and ACM have emerged independently, they appear to have more similarities than differences, providing considerable opportunity for cross-learning and exchange.

## Conclusion

Natural resource management, including conservation, is inherently complex and uncertain. The social and relational dimensions of social-ecological systems, including multiple and diverse stakeholders and their dynamic relationships with one another and the ecosystem in which they are embedded, make it especially challenging. Adaptive management has become the mantra for effective management, but there is considerable disenchantment about its utility and practicability. Our synthesis of SAM addresses an often-mentioned gap in adaptive management literature: examples of real-world adaptive management programs.

We used theory and hands-on experience to profile SAM, with three divergent streams adding richness. In SANParks, SAM benefitted from institutionalization and embedded researchers, which promoted resourcing, long-term science-management partnerships, reflexive practice and an ongoing operational example. The Australian stream represents a younger initiative largely driven by academics but with some institutional support and medium-term funding, with trials sometimes foundering. The Eastern Cape stream had to rely on academic champions and short-term project funding, and whether their SAM efforts can be sustained under these conditions will have to be seen. However, in their brief history, this stream developed progressive stakeholder engagement through participatory monitoring, evaluation, reflection and learning. Overall, experiences from these diverse streams indicate that SAM is flexible across contexts and scalable across applications, from local fire management experiments to conservation and restoration of ecosystems across large social-ecological landscapes.

Our case study also revealed many challenges, and we certainly do not claim perfect implementation. But the process is enabling, promoting co-learning and collaboration among diverse stakeholders, seeking to achieve an agreed future desired state. It balances the need for immediate action with a plan for structured learning and ongoing improvement, amidst uncertainty and resource constraints. It uses new information and insights from monitoring, directed research, the evolving evidence base and observations to review appropriateness and effectiveness of management interventions for informed adaptations.

We revisit our remark at the beginning, that there has been limited uptake of SAM in the formal adaptive management literature. Possibly, most SAM practitioners were more concerned with application than theorizing or marketing. More than 25 years of SAM practice has only produced 38 related articles through WoS and Scopus searches, with little marketing outside existing and relatively small streams of application led by a few key researchers (including the authors). Our comprehensive coverage of SAM will hopefully attract scrutiny from other adaptive management scholars and practitioners, and provide opportunity for knowledge exchange.

## Supplementary information

Supplementary information
